# rDromaserpin: A Novel Anti-Hemostatic Serpin, from the Salivary Glands of the Hard Tick *Hyalomma dromedarii*

**DOI:** 10.3390/toxins13120913

**Published:** 2021-12-20

**Authors:** Hajer Aounallah, Melissa Regina Fessel, Mauricio Barbugiani Goldfeder, Eneas Carvalho, Chaima Bensaoud, Ana Marisa Chudzinski-Tavassi, Ali Bouattour, Youmna M’ghirbi, Fernanda Faria

**Affiliations:** 1 Innovation and Development Laboratory, Innovation and Development Center, Instituto Butantan, São Paulo 05503-900, Brazil; hajer.aounallah@esib.butantan.gov.br (H.A.); melissa.fessel@butantan.gov.br (M.R.F.); mauricio.goldfeder@butantan.gov.br (M.B.G.); ana.chudzinski@butantan.gov.br (A.M.C.-T.); 2 Laboratory of Viruses, Vectors and Hosts (LR20IPT02), Pasteur Institute of Tunis, University of Tunis El Manar, Tunis 1002, Tunisia; ali.bouattour@pasteur.tn; 3 Laboratory of Bacteriology, Butantan Institute, São Paulo 05503-900, Brazil; eneas.carvalho@butantan.gov.br; 4 Institute of Parasitology, Biology Centre, Czech Academy of Sciences, 37005 Ceske Budejovice, Czech Republic; chayma.bensaoud@paru.cas.cz; 5 Centre of Excellence in New Target Discovery (CENTD), Butantan Institute, Butantã, São Paulo 05503-900, Brazil

**Keywords:** *Hyalomma dromedarii*, salivary glands, serpin, anticoagulants, thrombin inhibitor

## Abstract

Hemostatic disorders are caused either by platelet-related dysfunctions, defective blood coagulation, or by a combination of both, leading to an increased susceptibility to cardiovascular diseases (CVD) and other related illnesses. The unique specificity of anticoagulants from hematophagous arthropods, such as ticks, suggests that tick saliva holds great promise for discovering new treatments for these life-threatening diseases. In this study, we combined in silico and in vitro analyses to characterize the first recombinant serpin, herein called Dromaserpin, from the sialotranscriptome of the *Hyalomma dromedarii* tick. Our in silico data described Dromaserpin as a secreted protein of ~43 kDa with high similarities to previously characterized inhibitory serpins. The recombinant protein (rDromaserpin) was obtained as a well-structured monomer, which was tested using global blood coagulation and platelet aggregation assays. With this approach, we confirmed rDromaserpin anticoagulant activity as it significantly delayed plasma clotting in activated partial thromboplastin time and thrombin time assays. The profiling of proteolytic activity shows its capacity to inhibit thrombin in the micromolar range (0.2 to 1 μM) and in the presence of heparin this inhibition was clearly increased. It was also able to inhibit Kallikrein, FXIa and slightly FXIIa, with no significant effect on other factors. In addition, the rDromaserpin inhibited thrombin-induced platelet aggregation. Taken together, our data suggest that rDromaserpin deserves to be further investigated as a potential candidate for developing therapeutic compounds targeting disorders related to blood clotting and/or platelet aggregation.

## 1. Introduction

Despite the emergence of new diseases, cardiovascular diseases (CVD) such as stroke, heart attack, and deep vein thrombosis remain the leading causes of death worldwide [1], and are increasing exponentially given today’s modern unhealthy lifestyle [2]. They are multifactorial and are caused mainly by hemostasis disorders [3]. In a normal state, hemostasis serves to maintain the fluidity of blood within the veins and arteries, preventing excessive blood loss after injury through clot formation [4]. This system involves a set of well-regulated processes, including blood coagulation, platelet aggregation, and vasoconstriction, led by a complex series of cascade enzymatic reactions [5]. These reactions involve several serine proteases controlled by endogenous molecules including serine protease inhibitors (serpins) [6,7]. Several pathogeneses can ensue when serine protease activity or serpin-mediated regulation becomes unbalanced or dysfunctional [8]. For instance, a failure in the regulation of the blood coagulation may cause abnormal bleeding, causing either hemorrhage or thrombosis [9,10]. Anticoagulants and antiplatelet compounds are commonly used as antithrombotic drugs for the prevention and treatment of many cardiovascular disorders [11]. Apart from the CVD, some cases of severe thrombosis and vascular disorders have been related to the coronavirus disease 2019 (COVID-19) complications [12]. More recently, anticoagulant therapy has been applied to patients with coagulopathy associated with severe COVID-19, and the treatment has shown a decrease in the mortality rate [12]. Today’s treatment options for blood clotting dysfunction include naturally derived compounds [13]. With their high target specificity, they are not expected to have many side effects [13]. The search for alternative anticoagulants is still ongoing. Currently, several studies have screened natural resources, aiming to find potential antithrombotic biomolecules, worthy of competition or improvement to those currently available, with higher target specificity and/or lower side effects [14].

Serine protease inhibitors, termed serpins, are the most widely represented protein superfamily of protease inhibitors [15]. Admittedly, serpins have a pleiotropic function, regulating wide spectrums of proteolytic processes from thrombosis, thrombolysis, inflammation, and immune response, to cellular invasion in tissue remodeling, hormone transport, and even tumor growth and development [16]. Despite their multiple functions, serpins share a structurally conserved fold, including three β-sheets and seven to nine α-helices with a reactive center loop (RCL), recognized by target enzymes [17]. The RCL is a solvent-exposed adjustable stretch of 21–22 amino acid residues in length, which acts as a target for individual proteases [17]. Structural dynamic studies of serpins have unveiled the distinct conformational states adopted by the RCL, including the native intact RCL [18], the cleaved inactive form [19], the partially inserted RCL forms [20], and the latent form [21]. These studies have proved that the RCL must change its configuration to bind successfully to target proteases. While most serpins inhibit serine proteases, some of them inhibit cysteine proteases as well [17]. They function in a suicide inhibitory mechanism in which, once the serpin binds to the target protease, both molecules are permanently inactivated [15]. Through this inhibition, serpins toggle between normal physiology and pathology, guaranteeing normal biological function or leading to diseases, respectively [22]. Indeed, serpinopathies, where genetic mutations lead to inactive or aberrant serpins, and other disorders where serpin levels become unbalanced, cause thrombotic or thrombolytic cascades, triggering excess clotting or bleeding, respectively [23]. Restoring overall stability can be managed through the application of serpin protein treatments as therapeutics [24]. Given their central involvement, both in normal physiology and in pathological conditions, studies are increasing to better understand the roles of serpins in some physiological processes and to develop new therapeutic approaches.

Hematophagous animals, such as leeches, mosquitoes, and ticks employ serpins as a weapon to counter the host defense system and guarantee a successful blood meal [25]. These species represent an attractive natural source for the development of novel anti-hemostatic serpins. Particularly, proteins from tick salivary glands have been proven to target several blood coagulation components [26]. Several tick serpins have been described in numerous sequencing projects [27]; however, roughly a dozen of them are functionally described [28]. More specifically, to our knowledge, there is no previous characterization of serpins from the sialotranscriptome of *H. dromedarii.*

In the present study, we combined bioinformatics analyses and experimental assays to characterize a new serpin, named Dromaserpin, from the sialotranscriptome of *H. dromedarii.* Its recombinant form (rDromaserpin) was obtained by expression in *Escherichia coli*, was characterized using biochemical and biophysical tools, and tested in various in vitro assays. Its anticoagulant effect was demonstrated by its ability to significantly prolong the intrinsic coagulation pathway, acting primarily on thrombin and Kallikrein, two trypsin-like serine proteases central in the processes of hemostasis and thrombosis. Its antiplatelet activity was proven by inhibiting platelet aggregation induced by thrombin. Overall, our results suggest that rDromaserpin is a novel protease inhibitor that deserves further in-depth investigation, primarily its molecular and kinetic mechanism. Further in vivo studies could be the object of future studies initiating a path for the development of a new potential antithrombotic drug.

## 2. Results

### 2.1. Bioinformatic and Phylogenetic Analysis on Dromaserpin Sequence

**Figure 1:**
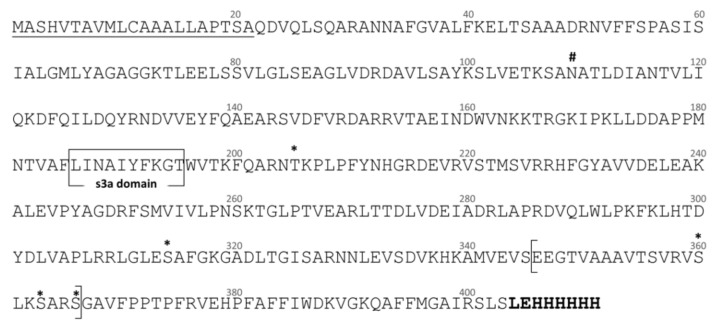
Analysis of the predicted amino acid sequence of Dromaserpin. The predicted signal peptide is underlined. N-glycosylation and O-glycosylation sites are identified by hashtag and asterisk, respectively. Conserved s3a domain is boxed and conserved reactive center loop (RCL) domain is indicated between square brackets. A poly-histidine tag and two residues, indicated in bold, were added to the C-terminal of the sequence. Numbers indicate amino acid residue positions in the sequence. The rDromaserpin sequence extends from residue 22 to residue 409. In the rDromaserpin, the signal peptide was removed and changed by a methionine.

The composite full-length Dromaserpin cDNA sequence was 1371 nucleotides long with a single open reading frame (ORF) of 1209 bases. The 5′ non-coding region was 27 bp, and the 3′ non-coding region was 135 bp (data not shown). The ORF coded for a protein with 403 amino acid residues, including a 21 residues signal peptide, which suggests the protein is secreted and acts extracellularly (Figure 1).

**Figure 2:**
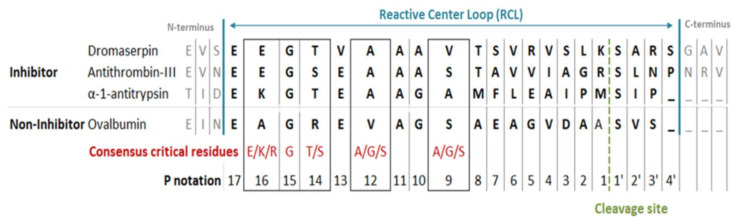
Comparison between RCL sequence in Dromaserpin and inhibitory and non-inhibitory serpins. RCL amino acid sequences alignment between α-Antitrypsin (Uniprot: P01009) and Antithrombin-III (P01008) from human, Ovalbumin from chicken (Uniprot: P01012), and Dromaserpin was obtained using ClustalW. The consensus critical residues for inhibitory activity have been described elsewhere [29]. P notation was applied according to a pervious study [30].

The predicted rDromaserpin has 391 amino acid residues, including the histidine tag sequence, with a theoretical molecular weight of 43,159.27 Da. The predicted protein has a single potential N-glycosylation site at the position N_109,_ and five O-glycosylation sites at the positions T_205_, S_314_, S_360_, S_363_ and S_366_ (Figure 1). The predicted protein presents the serine protease inhibitor-associated domains s3a and RCL (Figure 1) and, indeed, its membership in the serpin superfamily was confirmed by SMART software (Figure 1). Since the RCL consensus critical residues for serpin inhibitory activity were previously described [29], we aligned the predicted RCL of the newly identified serpin with the corresponding amino acid sequence of inhibitory and non-inhibitory serpins (Figure 2).

The RCL sequence extends from P17 to P4′. P′ residues are numbered from the cleavage site to the C-terminus. P residues are numbered from the cleavage site to the N-terminus. Dromaserpin’s RCL was similar to both inhibitory serpins α-1 Antitrypsin and Antithrombin-III, unlike the non-inhibitory serpin ovalbumin (Figure 2). Particularly, the consensus restudies described as crucial for the inhibitory activity were present in Dromaserpin among these: Glu_347_, Gly_348_, Thr_349_, Ala_351_, and were perfectly conserved, except Val_354_. Comparison of the aligned RCLs in Figure 2 hypothesizes that Dromaserpin may be an inhibitory serpin.

**Figure 3:**
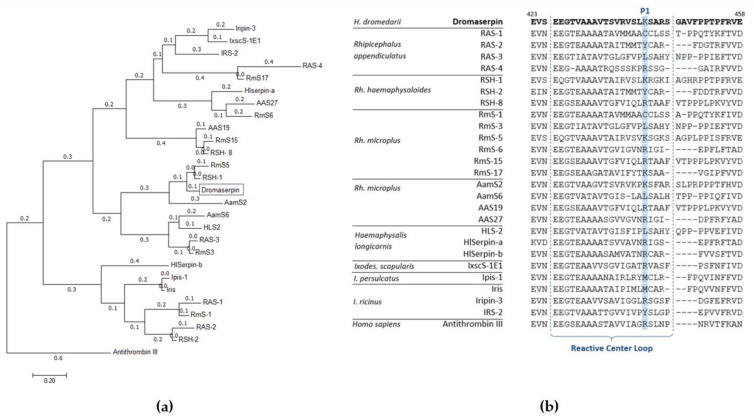
Molecular phylogenetic analysis of 25 tick serpins and Dromaserpin. (**a**) The evolutionary phylogenetic tree of Dromaserpin and the chosen tick serpins. Antithrombin III was utilized as an outgroup. The tree with the highest log likelihood (−12,179.3074) is shown, drawn to scale, with branch lengths measured in the number of substitutions per site (next to the branches). Evolutionary analyses were conducted in MEGA7 [33]. (**b**) Alignment of the RCL regions of the selected serpins was carried out using MEGA7.

As proteins sharing sequence similarities are quite likely to have similar or close activity [31], we aligned the Dromaserpin amino acid sequence with 25 tick serpins (Table S1). These serpins have >30% homology with the Dromaserpin and most have been experimentally proven to act as anti-hemostatic proteins [32]. Sequence comparison showed that amino acid patterns related to the inhibitory propriety (mainly the RCL domain and s3a domain) were present in Dromaserpin and were conserved among all the compared tick serpins (Figure 3b). In the evolutionary analyses, the resulting phylogenetic tree revealed two separate groups of serpins (Figure 3a). Dromaserpin was clustered together with the larger one, composed of 18 serpins (Figure 3a). Dromaserpin was closer to the serpins of genus *Rhipicephalus*. Indeed, the BLASTP search identified two tick serpins, from *Rhipicephalus* genus, whose sequences were very similar to the Dromaserpin sequence.

Dromaserpin shares the highest sequence identity 83.33% (99% coverage) with RHS-1 (accession: AFX65224.1), an anticoagulant serpin from *Rh. haemaphysaloides* presenting anti-chymotrypsin activity [32]. Dromaserpin presents 81.94% identity (97% coverage) with RmS5 (accession: AHC98656.1), from *Rh. microplus*, which has not been functionally characterized [34]. In the tree, Dromaserpin formed a small branch with RHS-1 and RmS5, both presenting a basic amino acid (lysine) in the position P1, similar to Dromaserpin (Figure 3a). The aligned RCLs of the serpins used in the phylogenetic analysis are described in (Figure 3b). The serpins grouped in the same cluster had highly conserved RCLs compared to other tick serpins, which showed fewer similarities.

### 2.2. Expression and Purification of the Recombinant Dromaserpin (rDromaserpin)

**Figure 4:**
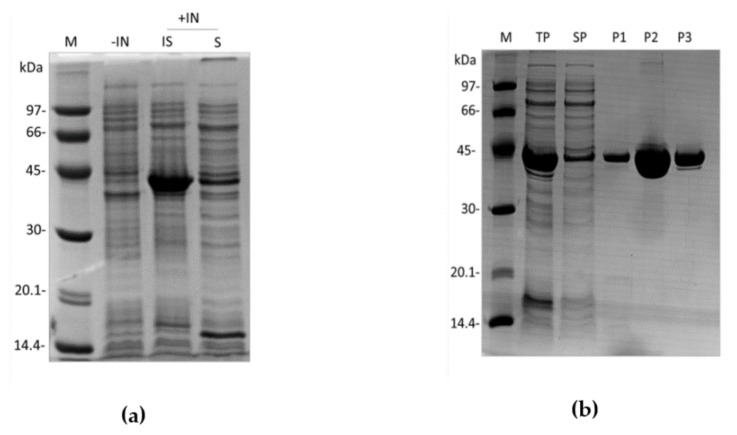
Expression and purification of rDromaserpin. cDNA coding for rDromaserpin was cloned into pET28a expression vector and the recombinant protein was expressed in *E. coli* BL21 (DE3) in 2YT medium. Whole cell lysates of non-induced or induced (IPTG 1 mM, 3 h, 30 °C) cultures were analyzed by SDS-PAGE (12.5%). (**a**) Both soluble and insoluble fractions were analyzed. Lanes M, −IN, +IN, IS, and S represent protein marker, not induced, induced, insoluble and soluble fractions, respectively; (**b**) SDS-PAGE of fractions from three chromatography steps. Lanes M, TP, and SP represent protein marker, total protein and soluble protein respectively. Lanes P1, P2, and P3 represent eluted purified proteins pooled from IMAC chromatography, Q sepharose ion-exchange chromatography, and size exclusion chromatography, respectively.

rDromaserpin has 391 amino acid residues, including the histidine tag sequence, a predicted molecular weight of 43,159.27 Da, and a predicted isoelectric point (pI) of 8.03. These features dictated the purification strategy adopted. rDromaserpin was successfully expressed using the *E. coli* BL21 (DE3) system in the optimal expression condition (1 mM IPTG, 30 °C, 3 h incubation). It was obtained in both soluble and insoluble forms (Figure 4a).

Although rDromaserpin mostly aggregates as inclusion bodies, it was possible to use the soluble fraction for the purification procedure and still obtain an acceptable yield (1.73 mg/L). As expected, purified rDromaserpin migrates at an apparent molecular weight of ~43 kDa on 12.5% SDS-PAGE (Figure 4b). After the purification steps, rDromaserpin was obtained with a high level of purity, observed after Coomassie Blue staining. The purified protein was used for subsequent experiments.

### 2.3. Structural Characterization of the rDromaserpin

#### 2.3.1. Analysis of the Secondary Structure of rDromaserpin

**Figure 5:**
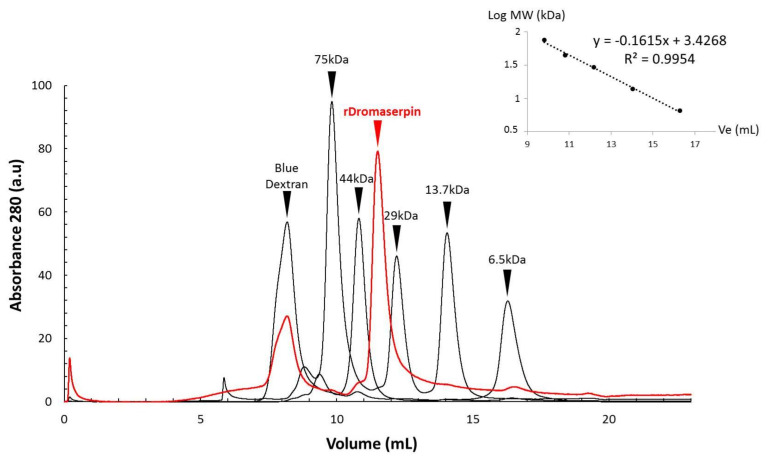
Separation of rDromaserpin and five standard proteins by size exclusion chromatography. Loading of the 100 μL of rDromaserpin (labeled in red) or the following standards (labeled in black): Conalbumin (75 kDa), Ovalbumin (44 kDa), Carbonic anhydrase (29 kDa), Ribonuclease A (13.7 kDa), Aprotinin (6.5 kDa), as well as blue dextran (for the void volume V_0_). A calibration curve was obtained with standards to calculate the molecular weight of rDromaserpin using the equation (log (MW) = −0.1615 Ve + 3.4268).

The purified rDromaserpin was obtained as a monomer, according to an analytical gel filtration analysis (Figure 5). According to the calibration curve obtained with standards, (Figure 5), the calculated molecular weight of rDromaserpin was 37.025 kDa.

**Figure 6:**
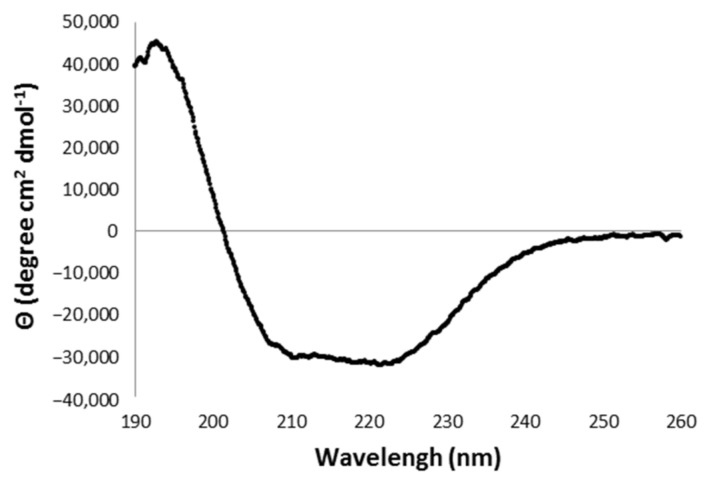
Far-UV CD spectra obtained for rDromaserpin. The ellipticity was expressed as the mean-residue molar ellipticity (θ) in degree cm^2^ dmol^−1^.

Its secondary structure content was investigated by carrying out circular dichroism (CD) analyses in the Far-UV region, which makes possible estimations of the protein’s α-helical, β-sheet and random coil content (Figure 6).

The Far-UV CD spectrum presented characteristics of a structured protein, and showed one positive peak at 194 nm and two minimums at 211 nm and 220 nm (Figure 6), a pattern related to an α/β rich protein [35]. The deconvolution of the CD spectrum, using the BestSel program, indicates the secondary structure content of rDromaserpin as approximately 54% α-helices, 21% β-strands and 25% random coils (Figure S1).

#### 2.3.2. Comparative Modeling

**Figure 7:**
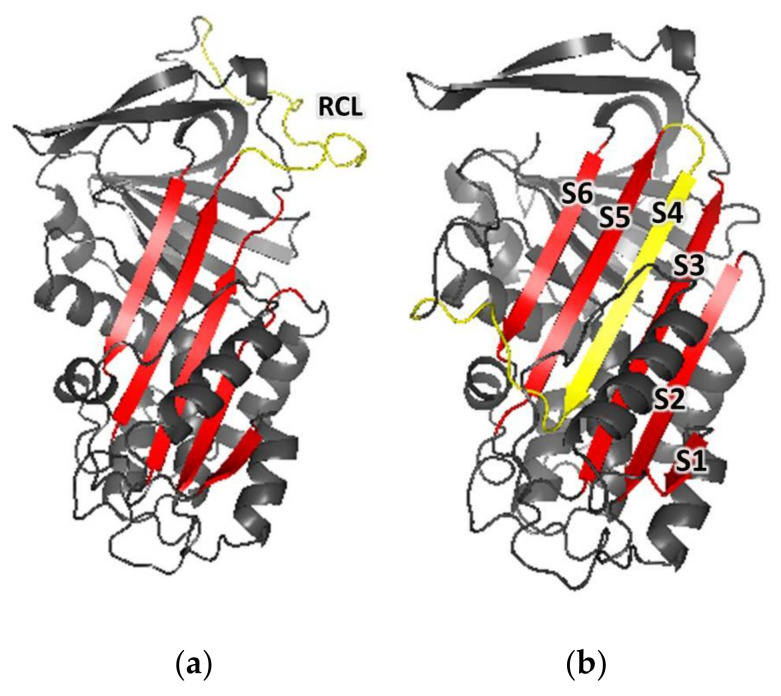
Cartoon representations of the three-dimensional models of Dromaserpin. (**a**) A cartoon representation of Dromaserpin 3D model 1 with an exposed RCL, based on the structure of Conserpin (PDB: 5cdx). (**b**) A cartoon representation of the Dromaserpin 3D model 2 with an inserted RCL, based on the structure of Conserpin (PDB: 5cdz). The RCL (in yellow) is inserted in the 5-sheet β-strand (in red). Loops are colored grey.

The predicted amino acid sequence of Dromaserpin was used to search for homologous proteins, with experimentally-solved 3D structures to serve as templates for structure homology modeling, using the Swiss-Model workspace. Conserpin, which shares 40.62% identity (92% coverage) with Dromaserpin, was the protein selected as a template, and had its 3D structure solved in both RCL open/uncleaved (PDB code: 5cdx) and closed/cleaved (PDB code: 5cdz) conformations. Two three-dimensional model structures of Dromaserpin were thus constructed using each of the conserpin conformations as templates (Figure 7).

According to the obtained models, the overall structure of Dromaserpin adopts a typical serpin fold composed of eight α-helices (Figure S2a) and three large β-sheets (A, B, and C) (Figure S2b). An uncleaved RCL model, based on the structure of Conserpin (5cdx), shows an intact RCL (Figure 7a) (yellow). As observed in other serpins [36], RCL in this configuration is located as a flexible loop outside the Dromaserpin structure core and can act as bait for target proteases. Additionally, a second model was built based on the structure of Conserpin in the latent state (PDB code: 5cdz) (Figure 7b). In this model, the RCL is cleaved and inserted into the central β-sheet A (Figure 7b) (S1, S2, S3, S5, and S6 indicated in red) as a strand, S4 (indicated in yellow on Figure 7b). Thus, according to Ramachandran plot, 97.6% (5cdz) (Figure S3) and 98.5% (5cdx) (Figure S4) of the residues from Dromaserpin 3D models are in favored and/or allowed regions. Neither model had residues located in the disallowed regions of the Φ, Ψ angle pairs of the Ramachandran plot, indicating correct stereochemistry. The percentage of secondary elements in Dromaserpin models was predicted by STRIDE, and was compared to those obtained experimentally by analyzing CD spectra, using the BestSel tool (Table 1).

### 2.4. Functional Characterization of the rDromaserpin

#### 2.4.1. Global Blood Coagulation Assays

**Figure 8:**
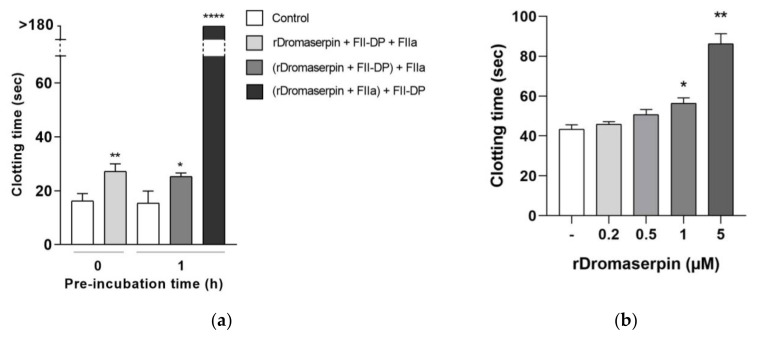
Effect of rDromaserpin on the intrinsic pathway of blood coagulation. (**a**) In vitro TT assay performed on FII-DP in the presence of 5 µM rDromaserpin and 2 mU FIIa. The assay was tested without pre-incubation or by pre-incubating samples in brackets for 1 h at room temperature. (**b**) In vitro APTT evaluation in isolated human plasma incubated for 20 min with 0.2, 0.5, 1, or 5 µM rDromaserpin. (-) refers to plasma used as control in the experiments containing the same volume of buffer. The results correspond to the mean ± SEM values acquired in three independent experiments; the asterisk indicates a significant difference between groups; * (*p* < 0.05) ** (*p* < 0.005) **** (*p* < 0.0005).

The effect of rDromaserpin was investigated on blood clotting using assays that independently target the extrinsic, intrinsic and common coagulation pathways. Prothrombin Time (PT) assay, designed to monitor the extrinsic coagulation [37], detected no significant effect upon the addition of 1 µM of rDromaserpin, and only a very slight increase of clotting time with 5 µM of rDromaserpin (2 s) in the tested conditions (data not shown). Thus, the protein, under these conditions, has minimal or no effect on the extrinsic coagulation pathway. As well, the Thrombin Time (TT) assay conducted on PPP (Platelet-poor plasma) did not show any significant differences under these conditions (data not shown). However, when tested with prothrombin deficient plasma (FII-DP), 5 µM of rDromaserpin significantly increased TT by ~10.97 s, when 2 mU of FIIa was added without pre-incubation (Figure 8a). Similarly, clotting time was increased by ~9.83 s, when rDromaserpin was pre-incubated with FII-DP for 1 h (Figure 8a). The plasma became incoagulable when rDromaserpin was pre-complexed with thrombin, preventing thrombin from converting the fibrinogen to fibrin (Figure 8a).

On the other hand, by using the activated partial thromboplastin time (APTT) assay, a statistically significant delay was shown in blood clot formation when plasma was treated during 20 min with 0.5, 1 or 5 µM rDromaserpin, prolonging the intrinsic coagulation time by 7.4 s, 13.06 s and 42.56 s, respectively (Figure 8b). Our results therefore suggest that rDromaserpin is probably able to inhibit serine protease(s) involved in the intrinsic pathway of blood coagulation.

#### 2.4.2. Protease Inhibition Profiling of rDromaserpin

**Figure 9:**
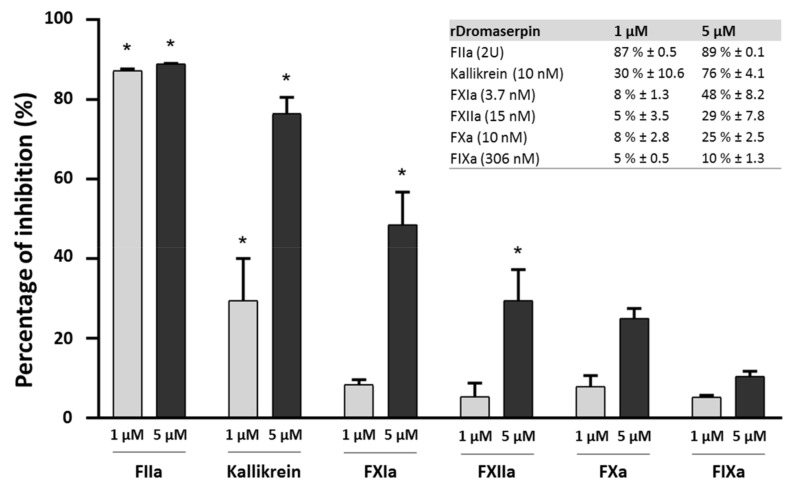
Inhibition of serine proteases by rDromaserpin. The ability of rDromaserpin (1 µM and 5 µM) to inhibit the activity of FIIa (2U), Kallikrein (10 nM), FXIa (3.7 nM), FXIIa (15 nM), FXa (10 nM), and FIXa (306 nM), using correspondent synthetic substrates (0.2 µM) was evaluated at 37 °C for 15 min. The results are acquired in three independent experiments. Proteases labelled with an asterisk were inhibited with statistical significance (*p* < 0.05).

As evidenced by its capacity to prolong the clotting time of human plasma, the ability of purified rDromaserpin to inhibit different serine proteases derived from human blood (FIIa, Kallikrein, FIXa, FXIa, FXIIa and FXa) involved in coagulation pathways was tested (Figure 9).

**Figure 10:**
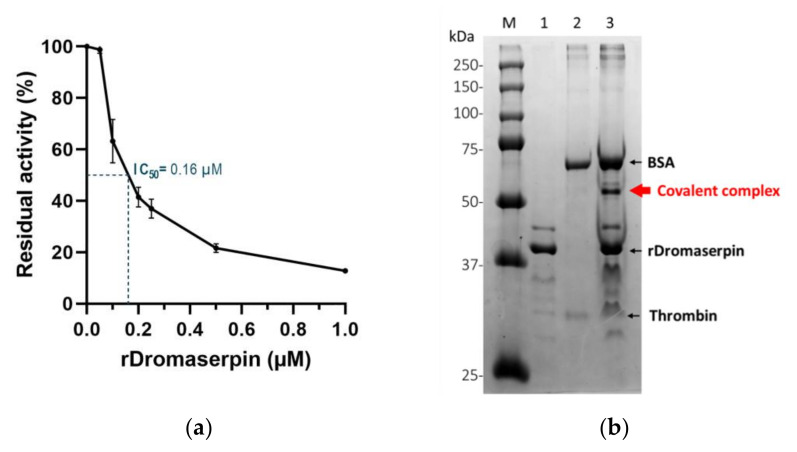
The effect of rDromaserpin on thrombin. (**a**) Residual activity of thrombin in the presence of increasing concentrations of rDromaserpin. Error bars represents the mean ± S.E.M values registered in three independent experiments. An estimate is given of the half maximal inhibitory concentration (IC_50_ = 0.16 µM). (**b**) A covalent complex was formed between rDromaserpin and thrombin. Lanes 1 and 2 represent rDromaserpin and thrombin, respectively. The major band in lane 2 corresponds to BSA (Bovine Serum Albumin) present in thrombin buffer. Lane 3 corresponds to rDromaserpin and thrombin loaded after 1 h incubation. Lane M represents the protein marker (Precision Plus Protein™ Dual Color Standars, BioRad). The covalent complex between rDromaserpin and thrombin is indicated with red arrow. Proteins were resolved on 5–10% SDS-PAGE and visualized by Coomasie Blue staining.

Residual proteolytic activity of the tested proteases was measured using the appropriate synthetic chromogenic substrates (see materials and methods section). Statistically significant reductions in enzymatic activity among the tested proteases were observed only for thrombin (FIIa), Kallikrein, and FXIa. At concentrations of 1 µM and 5 µM, rDromaserpin significantly decreased thrombin catalytic activity by 87% (*p* < 0.001) and 89% (*p* < 0.001), respectively. At the concentration of 5 µM, rDromaserpin significantly decreased Kallikrein catalytic activity by ~76% (*p* < 0.001), while 1 µM slightly inhibited the enzyme by ~30% (*p* < 0.01). Five µM of rDromaserpin inhibited FXIa (~48%, *p* < 0.01) and slightly FXIIa (~29%, *p* = 0.014). However, 1 µM of the inhibitor was unable to affect the activity of both factors. As opposed to FXa and FIXa which showed no significant activity in presence of rDromaserpin up to 5 µM. These data suggest that rDromaserpin is a functional thrombin inhibitor, able to target Kallikrein and FXIa, and it might play multiple roles in the tick-host interface. We further recorded thrombin activity using different concentrations of the inhibitor (ranging from 0.02 to 1 µM) (Figure 10a).

We observed a decrease in thrombin residual activity as the amount of rDromaserpin increased, inferring a dose-dependent inhibition (Figure 10a). At concentrations less than 0.1 µM, rDromaserpin did not affect thrombin (FIIa) activity. Indeed, it was able to significantly inhibit thrombin with an IC_50_ = 0.16 µM.

For a better understanding of its mechanism of action, we investigated the possibility of forming a covalent inhibitor-enzyme complex. With thrombin (36 kDa), rDromaserpin forms an SDS- and heat-resistant complex which migrates above 50 kDa (Figure 10b), proving a mechanism of suicide inhibition.

#### 2.4.3. Effect of Heparin on the Rate of Thrombin Inhibition by rDromaserpin

**Figure 11:**
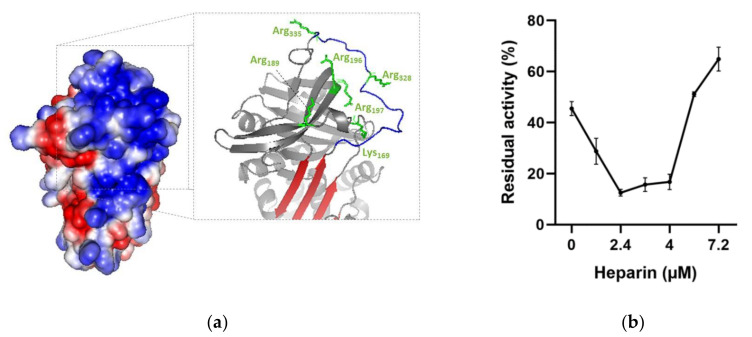

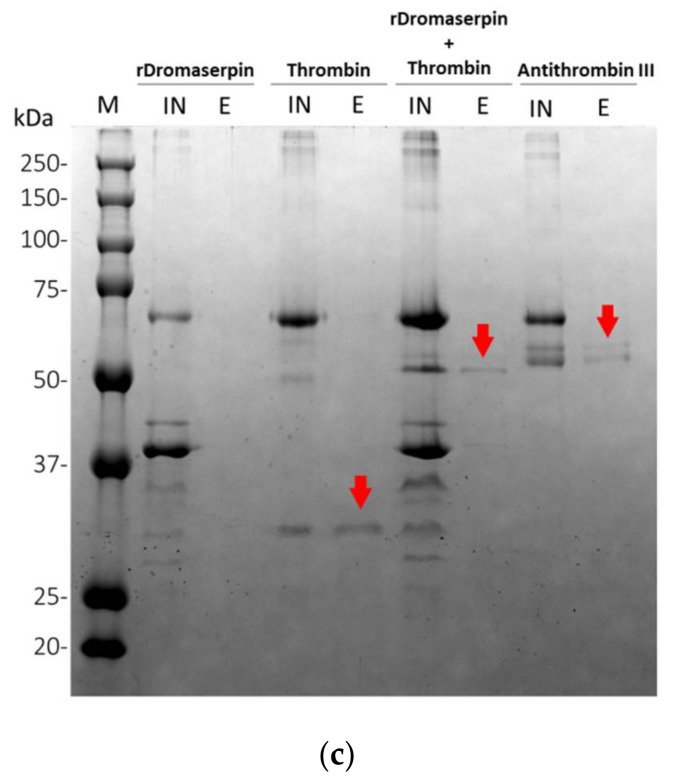
Effect of heparin on thrombin inhibition by rDromaserpin. (**a**) Electrostatic surface of Dromaserpin in the exposed RCL model. Colors relate to the electrostatic surface potential (blue is positive, and red is negative, −2 to 2 k_B_T) calculated by APBS (37). The surface charge distribution reveals a prominent basic patch located on top of the β-sheet A and in RCL region. Side chains of basic residues located and surrounding the basic path (Arg_196–197,_ Arg_189_, Arg_328_, Arg_335_ and Lys_169_) are labeled in green and shown as sticks. (**b**) Thrombin residual activity was assessed in the presence of 0.2 µM Dromaserpin and an increased concentration of heparin in µM range. Bar error represents the mean ± S.E.M values registered in three independent experiments. (**c**) Binding of rDromaserpin-thrombin complex to heparin. IN, input. E, eluted. Proteins eluted with 1 M NaCl are indicated by the red arrows. Antithrombin III was used as control, known to bind to heparin-sepharose resin.

Glycosaminoglycans are known to improve the inhibitory activity of plasma serpins [38]. We therefore investigated whether heparin, a common serpin activator, is able to enhance rDromaserpin activity toward thrombin. To locate the heparin binding site in the Dromaserpin model in its predicted active state (exposed RCL) (Figure 11a), we examined possible basic residues in revealed binding sites of other heparin-binding serpins. Our in silico analysis showed a single extended basic patch located on the top of the β-sheet A, near β-sheet C, and surrounding the RCL region in the active model of Dromaserpin (Figure 11a). Under the tested conditions, rDromaserpin did not bind to the heparin-sepharose resin (Figure 11c). In contrast, when it was pre-complexed with thrombin, the covalent complex was able to bind to the resin, indicating ternary complex formation (Figure 11c).

Experimentally, rDromaserpin induces accelerated thrombin inhibition in the presence of heparin, as evidenced by a U-shaped dose-response curve (Figure 11b). In the absence of heparin, thrombin was inhibited by 0.2 µM of rDromaserpin, showing a residual activity of 48.11% (Figure 11b). Using a range of heparin, the rate of inhibition appears to increase by around 25-fold in an optimal heparin concentration of 2.4 µM, reaching 12.5% of residual activity (Figure 11b). However, heparin-mediated acceleration of thrombin inhibition by rDromaserpin was progressively reduced upon a concentration of 5.6 µM (Figure 11b).

#### 2.4.4. Dromaserpin Inhibits the Function of Thrombin in Activating Platelet Aggregation

**Figure 12:**
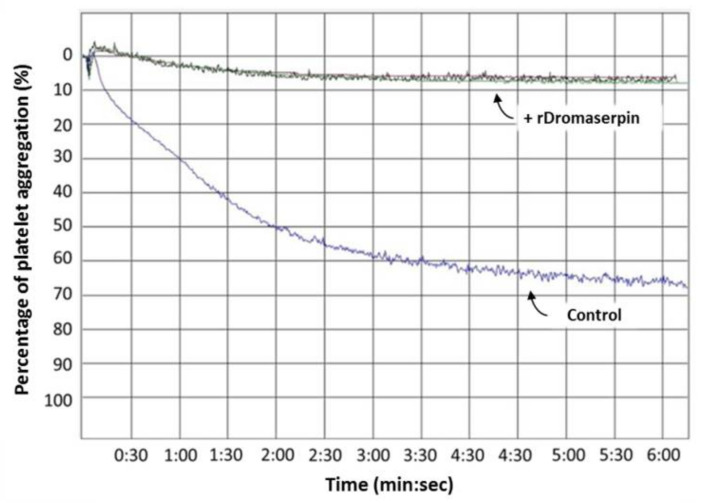
Effect of rDromaserpin on thrombin-induced platelet aggregation. The platelet aggregation assay was performed using washed platelet approach described in Material and Methods section. The trace below (labeled “control”) corresponds to platelet activated by thrombin in the presence of rDromaserpin buffer. The traces above (labeled “+ rDromaserpin”) correspond to triplicates of 10 µM rDromaserpin pre-incubated with thrombin prior to platelet activation. Data are presented as mean ± S.E.M. of triplicate platelet aggregation assays.

Platelet aggregation is one of the host’s first lines of anti-tick defense [5]. Knowing that ticks use serpins to hijack host hemostasis [39], and after discovering that rDromaserpin is an efficient inhibitor of thrombin in its action on blood clotting, it was necessary to verify whether it would also be able to inhibit platelet aggregation when thrombin is the agonist. Washed platelets were used in platelet aggregation assays in the presence of 1 μg/mL of thrombin as an agonist, and pre-incubated with 10 µM of rDromaserpin (Figure 12). Our results showed that rDromaserpin was able to inhibit the platelet aggregation function of thrombin by reducing platelet aggregation by 88.16% (±2.84%), compared to the control (Figure 12).

## 3. Discussion

Tick serpins attract considerable attention in the field of drug discovery and development because of their anti-hemostatic and immunomodulatory properties [27]. The current study was prompted by our previous investigations describing the sialome of *H. dromedarii* tick, highlighting a variety of putative proteins, including serpins, with potentially therapeutic features [40,41]. Our strategy was to combine computational and experimental analysis to characterize a new serpin that could target and modulate the hemostasis system.

Our bioinformatics analysis described Dromaserpin as a serpin of ~43 kDa and 403 amino acids long with a signal peptide, suggesting its secretion and extracellular activity in *H. dromedarii*. The inhibitory activity of serpins is mainly executed by its RCL domain [36]. Noting that the RCL is not the only region to predict inhibitory serpins, other consensus sequences that are well preserved during the evolution have been studied elsewhere [29]. Here, we focus on the RCL region as it is directly involved in the serpin’s inhibition mechanism. The RCL sequence in Dromaserpin is located near its C-terminus. It is composed of 21 amino acids, as are most serpins [15]. It is noteworthy that some serpins do not have classical inhibitory activity [15]. These non-inhibitory serpins contain the RCL domain, but there is no evidence that it is involved in their physiological activity. For instance, they may serve in hormone transport [42] and corticosteroid-binding globulin production [43]. Given this information, we found it important to compare RCL sequences in Dromaserpin, inhibitory and non-inhibitory serpins. The RCL of Dromaserpin shares the highest homology with inhibitory serpins α-1 Antitrypsin and Antithrombin-III. These in silico predictions lead us to believe that Dromaserpin might act as an inhibitory serpin, which we subsequently proved with functional assays. While revealing the first high-resolution structure of the serpin α-1 Antitrypsin, Engh et al. unveiled striking evidence about residues in RCL that are proposed to interact with target proteases [19]. Interestingly, Dromaserpin preserves residues essential for inhibitory activity (Figure 2). Moreover, the RCL sequence includes a cleavable reactive center, defined as the P1-P1′ bond, in which the protease cleaves and forms a covalent complex with the serpin [15]. Lys-Ser is the scissile P1-P1′sequence in Dromaserpin. Where the serpin’s target protease may not be accurately predicted based on their P1 amino acid residue [8], several previous studies have provided relevant information about the nature of P1 residue in relation to targeted protease [44,45]. Most related investigations report that serpins with inhibitory functions against trypsin or thrombin have polar basic (Arg and Lys) residues at the P1 site, whereas those with aromatic (Phe, Tyr and Trp) residues at the P1 site are more likely to inhibit chymotrypsin [8]. Dromaserpin has a lysine residue at the P1 site, identical to RHS-1 and RmS5, the closest serpins in the evolutionary tree, which inhibit chymotrypsin, thrombin and FXa [32]. Similar observations were reported for AAS19, which has Arg at the P1 site, and targets numerous protease including FXa, FXIIa, thrombin, tryptase, and chymotrypsin [46]. HLserpin-A has also a basic (Arg) in that position; however, it is reported to inhibit the serine proteases cathepsin G, and FXa, and the cystein proteases papain and Cathepsin B, with no effect on thrombin [47]. Experimentally, we proved that rDromaserpin can inhibit thrombin, Kallikrein and, slightly, FXIIa and FXIa, but neither FXa nor FIXa. Overall, we consider that in silico analyses are not sufficient to predict Dromaserpin targets. Apart from the P1-P1′ sequence, dynamic studies on serpins demonstrate that the RCL domain changes its state in order to bind to target proteases [48,49]. Using comparative modeling, we showed Dromaserpin in two conformations: an exposed RCL and an inserted RCL in the β sheet A. Both conformations have been described for other tick serpins in previous structural analyses [50,51]. In these analyses, they assume the use of a suicide inhibition mechanism to inhibit proteases. Experimentally, rDromaserpin forms an SDS and heat-stable complex, with thrombin proving a suicide inhibition mechanism. Apparently, thrombin cleaves the RCL of rDromaserpin in its P1 amino acid residue, forming the obtained covalent complex in the SDS-PAGE gel (Figure 11c). Through this inhibition mechanism Dromaserpin binds to thrombin, and both molecules are permanently inactivated.

To gain functional insight on this new serpin, a recombinant form (rDromaserpin) was overexpressed using an *E. coli* expression system, to prove its potential inhibitory activity in vitro. The feasibility of serpin interaction with targeted proteases depends on their 3D structure and conformation in the solution [16]. A serpin’s inhibition mechanism is defined by their ability to switch between distinct structural configurations and interplay between kinetic stability and thermodynamic instability [8]. As described above, we investigated, computationally, the conformational stability of Dromaserpin by predicting its tertiary structure. Here we consider that active rDromaserpin adopts the conformation of the model where the RCL is exposed to the solvent, ready to interact with the target protease. We have deduced from this model 29.83% α-helices, 30.65% β-strands and 39.52% random coils. CD spectroscopy was used for secondary structural content analysis of the predicted models and to determine the rDromaserpin conformation. According to CD data, the active rDromaserpin is well folded; however, the experimental secondary structural content findings differed slightly to that from the theoretical prediction. This discrepancy between the CD results and the model might be explained by the fact that the model was built based on only 372 residues, while CD measures consider the entire protein sequence (391 residues). In fact, 4.9% of the recombinant protein (19 residues: 9 C-terminal and 10 N-terminal) analyzed by CD, were not aligned during the modeling. Moreover, 3.8% of the model sequence (two inserts of 14 residues) were not modeled by Swiss-MODEL and were assigned as random coils by the software. These two percentages account for 8.7% of the residues randomly indicated as coil whereas they may be α helices. This assumption may explain the observed difference in our results.

**Figure 13:**
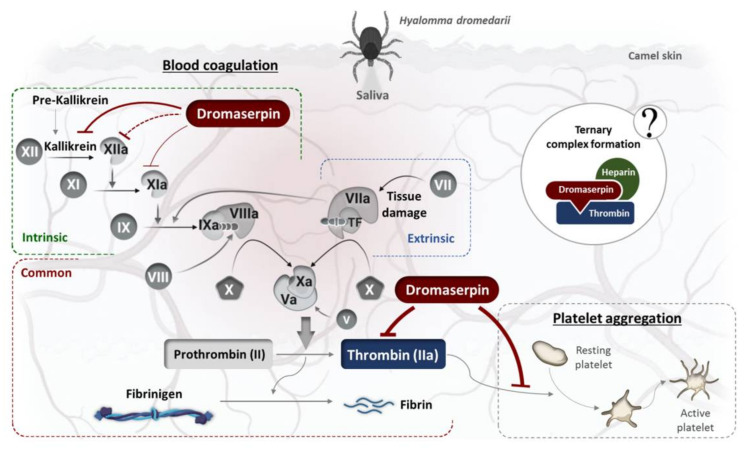
Schematic representation of rDromaserpin effect on hemostasis in the host interface.

Serpins generally function as serine proteinase inhibitors, hence their name. In mammals, serpins participate in the regulation of many complex proteolytic pathways [36]. Arthropod serpins are characterized by their hemostatic and anti-inflammatory effects in mammalian blood [52]. Accordingly, we investigated the implication of rDromaserpin in hemostasis. We first tested the effect of rDromaserpin on blood coagulation pathways. Initially, blood clotting was described as two converging enzymatic cascades, the extrinsic and the intrinsic coagulation pathways, stimulated either by exposure of blood to a damaged vessel surface or by blood-borne components of the vascular system, respectively [5]. Over the last century, major advances have been made in the field of hemostasis, promoting cell-based models of coagulation and explaining the hemostatic process as it occurs in vivo [53]. However, the cascade model has helped improve the understanding of coagulation in plasma-based in vitro assays and has allowed for clinically useful interpretations of laboratory tests for plasma coagulation anomalies [53]. We described our in vitro results using the cascade model to explain the involvement of rDromaserpin in blood clotting (Figure 13).

Here, we showed that rDromaserpin significantly prolonged the intrinsic pathway by 7.4 s, 13.06 s, and 42.56 s upon adding 0.5, 1, or 5 µM of inhibitor, respectively. In contrast, under the same condition, these amounts do not affect the extrinsic coagulation pathway (data not shown). In TT assay, the rDromaserpin prolonged the common coagulation pathway and significantly increased clotting time by ~10.97 s. It also increased clotting time by 9.83 s when pre-incubated with prothrombin deficient plasma. In contrast, the plasma became incoagulable when rDromaserpin was pre-incubated with thrombin. During the incubation time, the covalent complex formed and rDromaserpin prevented thrombin from converting fibrinogen to fibrin. We suggest, therefore, that rDromaserpin is likely to inhibit serine protease(s) involved in the intrinsic and the common pathway of blood coagulation. The anti-coagulation property of the camel tick salivary glands is already well documented [54]. Indeed, in an earlier investigation, five anticoagulants prolonging APTT were resolved from the salivary gland crude extract of *H. dromedarii* with no effect on PT [54]. However, the molecular identity of the main anti-coagulant molecules in *H. dromedarii* saliva is not fully known. Given its potential anticoagulant activity, we found it imperative to identify the rDromaserpin target(s) among certain factors involved in the intrinsic pathway of blood clotting. Interestingly, in vitro profiling of rDromaserpin’s proteolytic activity shows its capacity to inhibit thrombin and Kallikrein, to slightly affect FXIa and FXIIa activity without any relevant effect on FIXa or FXa. The intrinsic pathway is triggered by the activation of FXII when blood comes in contact with a negatively charged surface [6]. FXIIa (activated FXII) promotes its own activation in turn by stimulating Kallikrein formation and activating the upstream factor, FXIa [6]. Under the tested conditions, the effect of rDromaserpin on Kallikrein, FXIa, and FXIIa activity does not appear to be physiologically relevant. To verify its effectiveness in vivo, further experiments should be conducted under various conditions. Once these serine proteases (Kallikrein, FXIIa, and FXIa) are shown to be inhibited in vivo, coagulation time is prolonged, which might assist *H. dromedarii* in the initial stages of the feeding process. By targeting plasma Kallikrein, Dromaserpin could also help the tick avoid the formation of edema by inhibiting local bradykinin releases [55]. Nevertheless, it appears that Dromaserpin has a better efficacy on the common pathway of blood clotting, as it inhibits thrombin significantly. Indeed, among the serine proteases tested in this study, the strongest inhibition was observed with thrombin where the IC_50_ = 0.16 µM. In hemostasis, thrombin plays a key role in blood clotting, involved in the last steps of the common pathway, and in activating platelet aggregation. Indeed, due to its inhibitory activity on thrombin, we investigated whether rDromaserpin would affect platelet aggregation in addition to plasma clotting. Platelet aggregation is a complex process stimulated by several agonists including thrombin, cathepsin G, collagen and ADP [56]. Previous studies considered thrombin to be the most efficient platelet aggregation agonist [57,58]. In the present study, we showed that rDromaserpin significantly reduced platelet aggregation induced by thrombin.

Overall, these results suggest that Dromaserpin may play a crucial role during the tick fixation on the camel. Camels have a particularly active hemostatic mechanism with a short bleeding time and thrombocytosis [59]. They are known for their high level of FVIII in plasma (eight times more than humans), which is a very resistant factor to high temperatures [60]. This promotes hypercoagulability, where the intrinsic pathway is directly involved in high rates of thrombin generation. In this work, we present a tick molecule whose direct host is the camel. The camel’s parasites are likely to have powerful compounds in their saliva, such as Dromaserpin, that can keep its blood incoagulable to ensure successful feeding.

The exact mechanism of thrombin inhibition by Dromaserpin remains unclear. Nevertheless, Dromaserpin seems to damage their target protease after forming a stable complex, as proven with the rDromaserpin-thrombin covalent complex (Figure 11c). Usually, the visible consequence of this interaction is a dose-dependent reduction of the enzymatic activity of candidate proteases [61]. As a suicide inhibitor, rDromaserpin reduced residual enzyme activity of thrombin in a dose-dependent manner. On the other hand, serpin activity can be enhanced by cofactors, mainly glycosaminoglycans [38]. For several serpins, heparin interacts and modulates their activity by increasing the level of inhibition toward the targeted proteases [62]. Accordingly, we found it necessary to study whether heparin modulates the activity of Dromaserpin towards thrombin. Structurally, these interactions are mediated by defined and specific amino acid residues present in most heparin-binding proteins (BPH) [63]. In our in silico analysis, the Dromaserpin active model (exposed RCL) reveals an important basic patch located on top of the β-sheet A, near β-sheet C, and surrounding the RCL region. Indeed, electrostatic interactions play a major role in the binding process of heparin to serpins [63]. Molecular docking and computational approaches have revealed the usual consensus sequences, rich of basic residues, suitable to bind heparin [64,65,66]. As heparin is highly anionic, the basic amino acids, such as Arg_196-197,_ Arg_189_, Arg_328_, Arg_335_ and Lys_169_ (respecting the numbering in the model), in the region of the basic patch in Dromaserpin could be included in the heparin-binding site. It important to emphasize that these data are not sufficient to confirm the exact heparin-binding site in Dromaserpin, but the presence of this basic patch might support our hypotheses. Our preliminary investigations have shown that rDromaserpin alone is not able to bind heparin under the conditions tested. However, when complexed with thrombin, heparin-sepharose resin could capture the complex, proving the possibility of forming a ternary complex. The structural aspect of this interaction between rDromaserpin, thrombin, and heparin is under investigation.

The in vitro effect of heparin on thrombin inhibition by rDromaserpin was demonstrated by the increased inhibition rate in a certain range of heparin concentrations. Two mechanisms have been suggested to explain how heparin enhances protease activity and whether or not it binds to serpin only [38]. When only serpin binds to heparin, a saturation effect is usually observed with a range of heparin concentrations [38]. In contrast to our case, at high heparin concentrations, the curve obtained (Figure 11b) is probably consistent with the formation of a ternary complex (Figure 13). Heparin enhances several plasma serpins by using this template mechanism, such as SCCA1 [38], nexin-1 [67], and Antithrombin III [68]. By binding to antithrombin III, heparin acts as an anticoagulant, causing a conformational change in serpin which acts more effectively on coagulation factors and thrombin [68]. Similarly, a tertiary complex, including heparin, is probably formed when thrombin is inhibited by rDromaserpin. However, in-depth structural investigations are necessary to verify this hypothesis.

## 4. Conclusions

Gathering computational and experimental results, we were able to characterize a novel anti-hemostatic serpin, Dromaserpin, from the salivary glands of *H. dromedarii*. We believe that this is the first recombinant serpin identified from the *Hyalomma* genus which inhibits thrombin in both blood coagulation and platelet aggregation. We also suggested an inhibition mechanism implicating heparin as a cofactor. Future research will explore its molecular mechanisms and kinetics as an anticoagulant candidate. Besides, its effectiveness must be verified in vivo to testify its feasibility as a potential of medicinal applications mainly in treatment of blood clotting disorders.

## 5. Materials and Methods

### 5.1. Reagents and Chemicals

All chemicals and reagents were purchased from Sigma-Aldrich, Merck or Fisher, unless otherwise indicated, and were analytical grade or better. Human thrombin and factor Xa were purchased from Promega (Madison, WI, USA), human Kallikrein plasma was purchased from Sigma Aldrich (Saint Louis, MO, USA), and factor XIIa was purchased from HTI (Haematologic Technologies, Essex Junction, VT, USA). The activity of both factor XIa and factor IXa was tested using BIOPHENTM factor XIa (HYPHEN biomed, Neuville-sur-Oise, France) and BIOPHEN factor IXa, respectively. The chromogenic substrates S-2238 (H-D-Phe-Pip-Arg-pNA•2HCl) for thrombin, S-2765 (Z-D-Arg-Gly-Arg-pNA•2HCl) for factor Xa and S-2302 (H-D-Pro-Phe-Arg-pNA•2HCl) for FXIIa and Kallikrein were purchased from Chromogenix (Chromogenix, Milano, Italy). All solutions and buffers were prepared with Milli-Q water (Merck Millipore, Darmstadt, Germany).

### 5.2. Bioinformatics Analysis on Pedicted Dromaserpin Sequence

The sialotranscriptome of the *H. dromedarii* tick was recently described and annotated [41]. Among the deposited sequences, a cDNA sequence that codes for Dromaserpin was selected for expression. The nucleotide sequence was translated into an amino acid sequence using a TransDecoder, confirmed later with Expasy translate (https://web.expasy.org/translate/ (accessed on 1 April 2020)), and the predicted sequence was used for the following in silico analysis. A homologous search of the full-length Dromaserpin sequence was performed using BLAST programs (https://blast.ncbi.nlm.nih.gov/Blast.cgi (accessed on 5 October 2021)), through which the conserved domain was also identified. Localization of the identified RCL domain was done by SMART software (http://smart.embl-heidelberg.de/ (accessed on 1 April 2020)). Later, we performed a comparison between the RCL amino acid sequence in Dromaserpin and the RCL sequence of the inhibitory serpins α-1-antitrypsin (P01009) and Antithrombin-III (P01008), and a non-inhibitory serpin Ovalbumin (P01012), available in Uniprot. Full-length amino acid sequence was submitted to the signal 4.1 server (http://www.cbs.dtu.dk/services/SignalP-4.1/ (accessed on 1 April 2020)) and the signal peptide was removed from the protein sequence for subsequent analysis. The predicted molecular weight (MW) and isoelectric point (pI) were determined using ProtParam tools from ExPASy server (https://web.expasy.org/protparam/ (accessed on 1 April 2020)). To determine potential N- or O-linked glycosylation sites, Dromaserpin amino acid was scanned using NetNGlyc 1.0 (http://www.cbs.dtu.dk/services/NetNGlyc/ (accessed on 1 April 2020)) and NetOGlyc 4.0 servers (http://www.cbs.dtu.dk/services/NetOGlyc/ (accessed on 1 April 2020)). For phylogenetic analysis, protein sequences of 25 serpins from tick species and one human serpin were retrieved from GenBank. Accession numbers of these sequences, the percentage of identity and coverage with Dromaserpin are described in Supplementary Table S1. Multiple sequence alignments were performed using ClustalW algorithm (https://www.genome.jp/tools-bin/clustalw (accessed on 5 October 2021)) and sequences were edited manually. The evolutionary history was inferred by using the Maximum Likelihood method based on the JTT matrix-based model [69]. Human Antithrombin III was utilized as outgroup to root the tree. The initial tree was obtained automatically by applying Neighbor-Join and BioNJ algorithms to a matrix of pairwise distances, estimated using a JTT model, and then selecting the topology with superior log likelihood value. The tree is drawn to scale, with branch lengths measured in the number of substitutions per site. Evolutionary analyses were conducted in MEGA7 [33].

### 5.3. Plasmid Construction-Expression and Purification of the Recombinant Dromaserpin

All manipulation of microorganisms was developed in the BSL-2 area, as authorized by CIBio-IBu (Internal Biosafety Committee of Butantan Institute, Brazil) and CTNBio (National Technical Biosafety Commission, Brazil) (Registration number CQB-039/98 of 02/13/2014). The chosen full coding cDNA sequence was used for plasmid construction after the deletion of the sequence coding for signal peptide. Codon optimization, gene synthesis and molecular cloning of rDromaserpin coding sequence into plasmid pET28a (Novagen/EMD Millipore, Darmstadt, Hesse, Germany) were conducted by GenOne (GenOne Inc. Rio de Janeiro, RJ, Brazil). Recombinant pET28a_Dromaserpin plasmid, coding for the C-terminal His-tagged protein, was transformed into chemically competent *E. coli* BL21 (DE3) strain. The *E. coli* cultures were inoculated in 1 L of 2 YT culture medium (supplemented with 20 µg/mL kanamycin) at 30 °C with 250 rpm agitation. Protein expression was induced with 1 mM isopropyl β-D-Thiogalactoside (IPTG), at OD_600_ 0.6, and cells remained incubated for 3 h at 30 °C following the induction. Expression was confirmed by resolving samples on a 12.5% SDS-PAGE. Bacterial cells were harvested, washed with 150 mM NaCl and re-suspended in lysis buffer (50 mM Tris-HCl pH = 8; 500 mM NaCl, 1% Triton; 10 mM β-mercaptoethanol). Whole cell extracts were obtained through a Panda Plus 2000 (GEA NIRO, Erie, PA, USA) high pressure homogenizer disrupter, three times at 1000 bars, and the suspension was clarified through centrifugation at 16,000× *g* rpm for 1 h at 4 °C. Histidine tagged protein was subsequently purified from the soluble fraction using a HisTrap HP (5 mL; GE Healthcare, GE Healthcare, Uppsala, Uppland, Sweden) column of Immobilized Metal Affinity Chromatography (IMAC) (AKTA AVANT; GE Healthcare GE Healthcare, Uppsala, Uppland, Sweden). Fractions containing the eluted protein were pooled and subjected to Q-sepharose ion-exchange chromatography (AKTA AVANT; GE Healthcare, GE Healthcare, Uppsala, Uppland, Sweden) using a HiTrap Q FF (1 mL; GE Healthcare, GE Healthcare, Uppsala, Uppland, Sweden) column. The purified protein was applied to a Superdex 75 (1 mL; GE Healthcare, GE Healthcare, Uppsala, Uppland, Sweden) column used to exchange the buffer to 1 × Phosphate-buffered saline (PBS), pH 7.4 supplied with 10% glycerol. To evaluate its purity, the purified protein was resolved on a 12.5% SDS-PAGE and stained with Coomassie Brilliant Blue. Finally, rDromaserpin concentration was determined by its absorbance at 280 nm using a Biodrop spectrophotometer (Biochrom™ BioDrop μLite Micro-Volume, Braeside, Australia).

### 5.4. Structural Characterization of the rDromaserpin

#### 5.4.1. Determination of Molecular Size by Gel Filtration

Gel Filtration experiments were performed with purified rDromaserpin using Superdex™ 75 HR 10/300 column (GE Healthcare, Uppsala, Uppland, Sweden) on an ÄKTA purifier liquid-chromatography system. Chromatography was carried out at 4 °C in 1× PBS pH 7.4 and 10% glycerol, at a flow of 0.5 mL/min. Protein elution was monitored by measuring absorbance at 280 nm. Apparent molecular masses of protein eluted from the column were deduced from a calibration curve obtained by loading 100 μL of the following standards: Conalbumin (75,000 Da), Ovalbumin (44,000 Da, 30.5 Å), Carbonic anhydrase (29,000 Da), Ribonuclease A (13,700 Da, 16.4 Å), Aprotinin (6500 Da), as well as blue dextran (for the void volume V_0_). According to the calibration curve obtained with standards, we calculated the molecular weight of rDromaserpin as described in the Equation (1).

log(MW) = −0.1615 Ve + 3.4268(1)

#### 5.4.2. Circular Dichroism (CD) Spectroscopy

CD spectra were recorded on a JASCO J-810 spectropolarimeter, equipped with a thermoelectric sample temperature controller (Peltier system) equilibrated to 20 °C. The rDromaserpin samples at 0.73 µM were diluted 10 × in Milli-Q water. The diluted buffer without protein was used to calibrate the equipment. The scans were collected at Far-UV region (from λ = 190 nm to λ = 260 nm) after seven accumulations, using a pathlength quartz cuvette of 1.0 mm. Spectra were corrected by subtracting a buffer blank, normalized to residue molar absorption, measured in mdeg (M^−1^ cm^−1^), and adjusted to the input buffer. The mean molar residual ellipticity θλ (deg cm^2^ dmol^−1^) was calculated as described in the Equation (2), based on a molecular mass of 43,159.27 Da, where $\theta_{\lambda }^{Raw}$ is ellipticity in millidegrees, C is molar rDromaserpin concentration in M, N is number of amino acid residues, and l is the path length of the cuvette in mm.

(2) $$\theta_{\lambda }=\frac{\theta_{\lambda }^{Raw}\times 10^{6}}{C\times N\times L}$$

The estimation of secondary structure was performed by BeStSel website algorithm (http://bestsel.elte.hu/index.php (accessed on 9 April 2021)) using the CD spectra values ranging from 190 nm to 250 nm.

#### 5.4.3. Comparative Modeling and Structural Analysis of Dromaserpin

The three-dimensional structure (3D) model of Dromaserpin was predicted using a comparative modeling approach. Using its predicted amino acid sequence, we performed a homology search among the 3D structures available in PDB database (https://www.rcsb.org/ (accessed on 22 March 2020)) using BLAST from NCBI (https://blast.ncbi.nlm.nih.gov/Blast.cgi (accessed on 22 March 2020)). For homology modeling, we selected the crystal structure of Conserpin in an uncleaved (PDB code: 5cdx) and a latent (PDB code: 5cdz) state, which presents 40.62% identity with Dromaserpin, covering 94% of the sequence. Aligned regions were selected using 5cdx as template on Swiss-Model online tool (https://swissmodel.expasy.org/ (accessed on 22 March 2020)). A 3D model was generated by PyMOL software (http://www.pymol.org/ (accessed on 22 March 2020)). Subsequently, the overall quality of the Serpin 3D-structure models was evaluated by analyzing each correspondent Ramachandran plot, calculated by MolProbity program (http://molprobity.biochem.duke.edu/ (accessed on 30 September 2021)). From these generated models, we determined the content of the secondary structure using Stride (http://webclu.bio.wzw.tum.de/cgi-bin/stride/stridecgi.py (accessed on 22 March 2020)).

### 5.5. Functional Characterization of the rDromaserpin

#### 5.5.1. Global blood Coagulation Assays

All experimental procedures were conducted as per the guidelines for the care and use of laboratory subjects of the Butantan Institute. The study on human blood was approved by the National Human Research Ethics Committee, Plataforma Brasil, CAAE: 33013220.4.0000.0086. Informed consent was obtained from the volunteers conferring to the declaration of Helsinki Convention and the Brazilian Department of Health. Blood was drawn from healthy human volunteers and collected in a test tube preloaded with 3.2% sodium citrate. Plasma was harvested immediately by centrifugation of blood at 3000× *g* rpm at 19 °C for 15 min. APTT, PT, and TT assays were performed by incubating rDromaserpin with the plasma for 20 min as described elsewhere [70]. These assays were performed using commercially available kits (STA-PTT Automate 5 for APTT, STA- Neoplastine^®^ CI Plus for PT and STA-Thrombin 2 for TT–STAGO, Asnières-sur-Seine, France), using Semi-automatic coagulation analyzer START STAGO^®^. Alternatively, TT assay was also performed using FII-DP (Haematologic Technologies, Essex Junction, VT, USA). TT was measured after pre-incubating rDromaserpin with the plasma or with 2 mU thrombin for 1 h at room temperature. Clotting times were determined in triplicate in the absence or presence of 0.2, 0.5, 1, and 5 µM of rDromaserpin for APTT assay and 1 and 5 µM of rDromaserpin for PT and TT assays.

#### 5.5.2. Protease Inhibition Assays

Inhibitory activity of rDromaserpin was tested against six mammalian serine proteases related to host defense pathways against tick feeding. The mammalian proteases tested (per reaction) included: human factor XIIa (15 nM), human thrombin (2U), human factor Xa (10 nM), human plasma Kallikrein (10 nM), human factor XIa (3.7 nM) and human factor IXa (306 nM) (Enzyme Research Laboratories). Substrates were used at 0.2 mM final concentration, including S-2238 for thrombin, S-2302 for factor XIIa and Kallikrein, and substrate S-2765 for factor Xa. Reagents were mixed at room temperature in technical triplicates. Two concentrations of rDromaserpin (1 μM and 5 μM) were pre-incubated separately with indicated amounts of the proteases listed above for 15 min at 37 °C in 20 mM Tris-HCl, 150 mM NaCl, BSA 0.1%, pH 7.4 buffer. The corresponding substrate for each protease was added in a 100 μL final reaction volume, and substrate hydrolysis was measured at OD_405_ nm every 11 s for 15 min at 37 °C using Epoch™ Microplate Spectrophotometer (BioTeck). The inhibitory activity on factor XIa and factor IXa was tested according to the guidelines of their correspondent kits (HYPHEN biomed, Neuville-sur-Oise, France). Acquired OD_405_ nm data are subjected to one-phase decay analysis in Prism 8 software to determine plateau values as proxies for initial velocity of substrate hydrolysis Vmax or residual enzyme activity. The percent enzyme activity inhibition level is determined using the Equation (3), where $Vmax_{Vi}\;$ is activity in presence of rDromaserpin and $Vmax_{V0}\;$ is activity in absence of rDromaserpin. Data are presented as mean ± S.E.M of three independent replicate readings.

(3) $$100-(\frac{Vmax_{Vi}}{Vmax_{V0}})\;\times \;100$$

As thrombin was the most strongly inhibited protease, its activity was later tested with rDromaserpin at different concentrations (1, 0.5, 0.25, 0.2, 0.1, 0.05, and 0.02 µM) using the protocol described above. The covalent complex was visualized by incubating 1.7 µM rDromaserpin and 2U Thrombin for 1 h at room temperature. After one hour of incubation, 10 µL of 5 × loading buffer (62.5 mM Tris –HCl pH 6.8, 3% SDS, 10% glycerol, 1.25% β-mercaptoethanol, 0.001% blue of bromophenol) were added to the mixture, and samples were boiled for 10 min. Finally, samples were resolved on a 5–10% SDS-PAGE and stained with Coomassie Brilliant Blue.

#### 5.5.3. Thrombin Inhibition by rDromaserpin in the Presence of Heparin

For assays in the absence or presence of heparin, the concentration of thrombin was held at 2U, and the concentration of rDromaserpin was maintained at 0.2 µM. The stock of unfractionated heparin concentration of 10 µg/mL used in this assay represents a concentration of ~400 µM (taking an average molecular mass of 25,000 g/mol). Heparin was added to the reaction in a range of 0.8–7.2 µM. Reactions were incubated for 15 min at 37 °C in thrombin assay buffer. The chromogenic substrate S-2238 was used at a final concentration of 0.2 mM, and residual activity was followed at OD_405_ nm.

#### 5.5.4. Evaluation of Binding to Heparin-Sepharose Resin

The ability of rDromaserpin, thrombin and the covalent complex to bind to heparin was evaluated using Heparin-Sepharose 6 Fast Flow affinity resin (Cytiva, Marlborough, MA, USA). First, 50 µL of the resin was equilibrated with 200 µL of buffer A (20 mM Tris pH 7.4, 150 mM NaCl, 0.1% BSA). In four different tubes, 32 µg rDromaserpin, 133 U thrombin, rDromaserpin-thrombin complex, or 50 µg antithrombin III were added to the resins in a final volume of 250 μL. The samples were incubated for 30 min with gentle shaking. The resins were washed three times with 200 µL of buffer A to remove unbound proteins. For elution, 1 M NaCl was added and incubated for 10 min. The resins were pelleted by centrifugation at 1000× *g* rpm for 5 min, and the supernatants were analyzed by 10% SDS-PAGE.

#### 5.5.5. Effect of rDromaserpin on Platelet Aggregation

The effect of rDromaserpin on the function of thrombin in platelets was also investigated on washed platelets. To prepare washed platelets, approximately 20 mL of freshly human blood collected from healthy donors, in a tube containing 3.8% sodium citrate, was centrifuged at 900× *g* rpm for 20 min at room temperature. Platelet rich plasma (PRP) was recuperated and 2% EDTA was added at a ratio of 1:100, followed by 2000 rpm centrifugation for 15 min at room temperature. The pellet containing platelets was resuspended in 10 mL of wash buffer (140 mM NaCl, 10 mM NaHCO_3_, 2.5 mM KCl, 0.49 mM Na_2_HPO_4_, 1 mM MgCl_2_, 22 mM Sodium Citrate, 52.23 mM BSA-dissolved in 100 mL of water, pH adjusted to 6.5 with HCl), and centrifuged at 2000× *g* rpm for 15 min at room temperature. The wash step was repeated twice under the same conditions. The supernatant was discarded and the pellet resuspended in 2 mL of Tyrode buffer (1 mM CaCl_2_, 134 mM NaCl, 12 mM NaHCO_3_, 2.9 mM KCl, 0.34 mM Na_2_HPO_4_, 1 mM MgCl_2_, 10 mM HEPES-pH adjusted to 7.4 with HCl. Add glucose 1% at the time of use). The aggregometer (CHRONO-LOG^®^ Model 700, Havertown, PA, USA), was calibrated with Tyrode buffer containing glucose. To determine the effect of rDromaserpin on platelet aggregation, 10 μM rDromaserpin or equal volume of 1 × PBS buffer pH 7.4, 1, 10% glycerol were pre-incubated for 10 min at 37 °C, with agonist 1 μg /mL NIH-U thrombin in a 50 μL reaction. Platelet aggregation was induced following the addition of pre-warmed washed platelets in a final volume of 500 μL. Platelet aggregation was monitored for 6 min and the percentage of platelet aggregation inhibition was deduced using The AGGRO/LINK^®^8 program. Data are presented as mean ± S.E.M. of triplicate platelet aggregation assays.

### 5.6. Statistical Analysis

Data are presented as the mean ± S.E.M unless indicated otherwise and statistical significance was calculated for at least three independent experiments employing the Student’s *t*-test, using GraphPad Prism 8.0 software (GraphPad Software, Inc., San Diego, CA, USA). *p*-values < 0.05 were considered to be statistically significant.

## Figures and Tables

**Table 1:** Comparison of the secondary structure content obtained theoretically (exposed RCL and inserted RCL) and experimentally (CD data).

	Elements from the Models		Elements from CD Data
	Exposed RCL	Inserted RCL	
Total aligned residues (100%)	372	372	391
Helix (%)	29.83	30.11	54.20
Strand (%)	30.65	33.60	21.40
Other (Coil, bridge, Turn) (%)	39.52	36.29	24.40

## Supplementary Materials

The following are available online at https://www.mdpi.com/article/10.3390/toxins13120913/s1. Table S1: GenBank accession numbers of serpins used in the phylogenetic analysis; Figure S1: CD spectrum analysis by the BesSel program; Figure S2: Cartoon representations of the secondary elements in the active model of Dromaserpin; Figure S3: Ramachandran plot of Dromaserpin Model with an exposed RCL, generated by MolProbity program; Figure S4: Ramachandran plot of Dromaserpin Model with an inserted RCL, generated by MolProbity program. References [32,34,47,50,71,72,73,74,75,76,77,78] are cited in the supplementary materials.
