# Supplementary material for: rDromaserpin: A Novel Anti-Hemostatic Serpin, from the Salivary Glands of the Hard Tick Hyalomma dromedarii
Source: Toxins (Basel). 2021 Dec 20;13(12):913. doi: 10.3390/toxins13120913 (PMC8703697; doi:10.3390/toxins13120913)
Supplement: Supplementary file 1 [file toxins-13-00913-s001.zip › toxins-1461751-supplementary.pdf]

toxins

MDPI

Supplementary Material: rDromaserpin: A Novel Anti-Hemostatic Serpin, from the Salivary Glands of the Hard Tick *Hyalomma dromedarii*

Hajer Aounallah, Melissa Regina Fessel, Mauricio Barbugiani Goldfeder, Eneas Carvalho, Chaima Bensaoud, Ana Marisa Chudzinski-Tavassi, Ali Bouattour, Youmna M'ghirbi and Fernanda Faria

Table S1. GenBank accession numbers of serpins used in the phylogenetic analysis.

Serpin	Tick species	Accession number	% identity	% Coverage	References
Antithrombin III	*Homo sapiens*	AAB40025.1	31.32	92	[71]
AamS2	*Amblyomma americanum*	ABS87354.1	60.71	95	[72]
AamS6	*Amblyomma americanum*	ABS87358.1	45.87	92	[72]
AAS19	*Amblyomma americanum*	JAI08902.1	38.13	97	[73]
AAS27	*Amblyomma americanum*	JAI08961.1	39.84	92	[73]
HLS2	*Haemaphysalis longicornis*	BAD11156.1	45.59	98	[73]
HlSerpin-a	*Haemaphysalis longicornis*	QFQ50847.1	39.84	92	[74]
HlSerpin-b	*Haemaphysalis longicornis*	QFQ50848.1	33.69	91	[47]
Ipis-1	*Ixodes persulcatus*	BAP59746.1	37.07	91	[47]
Iripin-3	*Ixodes ricinus*	JAA69032.1	41.33	92	[75]
Iris	*Ixodes ricinus*	CAB55818.2	37.33	91	[76]
IRS-2	*Ixodes ricinus*	ABI94056.2	39.75	99	[50]
IxscS-1E1	*Ixodes scapularis*	AID54718.1	40.27	92	[77]
RAS-2	*Rhipicephalus appendiculatus*	AAK61376.1	33.24	87	[78]
RAS-1	*Rhipicephalus appendiculatus*	AAK61375.1	35.2	91	[78]
RAS-3	*Rhipicephalus appendiculatus*	AAK61377.1	45.06	97	[78]
RAS-4	*Rhipicephalus appendiculatus*	AAK61378.1	30.45	98	[78]
RmS-1	*Rhipicephalus microplus*	AHC98652.1	35.64	91	[34]
RmS3	*Rhipicephalus microplus*	AHC98654.1	47.73	95	[34]
RmS5	*Rhipicephalus microplus*	AHC98656.1	81.94	97	[34]
RmS-6	*Rhipicephalus microplus*	AHC98657.1	38.37	97	[34]
RmS-15	*Rhipicephalus microplus*	AHC98666.1	39.68	92	[34]
RmS-17	*Rhipicephalus microplus*	AHC98668.1	40.15	98	[34]
RHS-1	*Rhipicephalus haemaphysaloides*	AFX65224.1	84.51	97	[32]
RHS-2	*Rhipicephalus haemaphysaloides*	AFX65225.1	34.52	93	[32]
RHS-8	*Rhipicephalus haemaphysaloides*	QHU78941.1	38.9	98	[32]

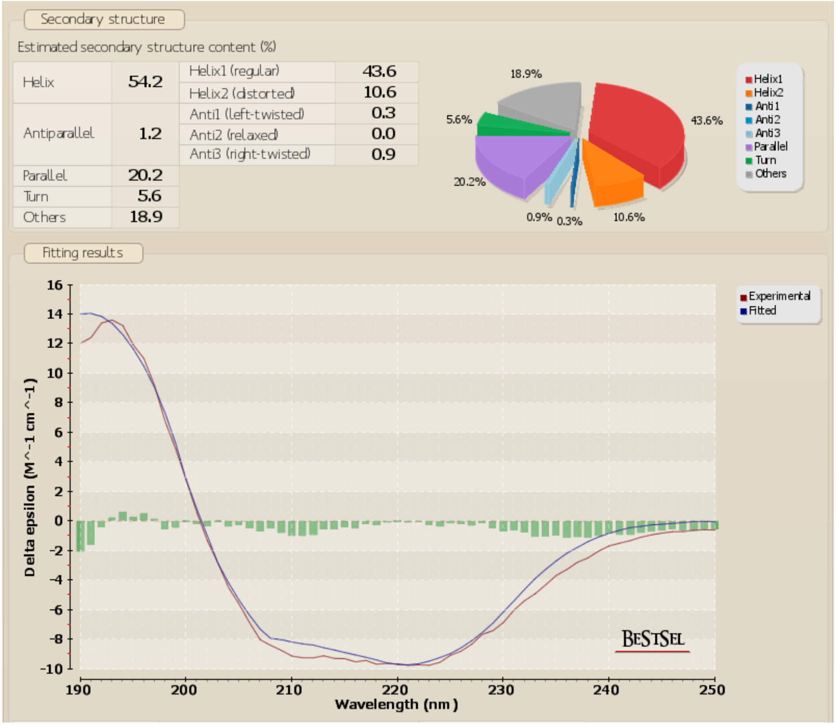

Figure S1. Circular dichroism (CD) spectrum analysis by the BesSel program. Above is described the estimated secondary structure content (%) in rDromaserpin. Below are shown the experimental and predicted CD spectrum by BestSel.

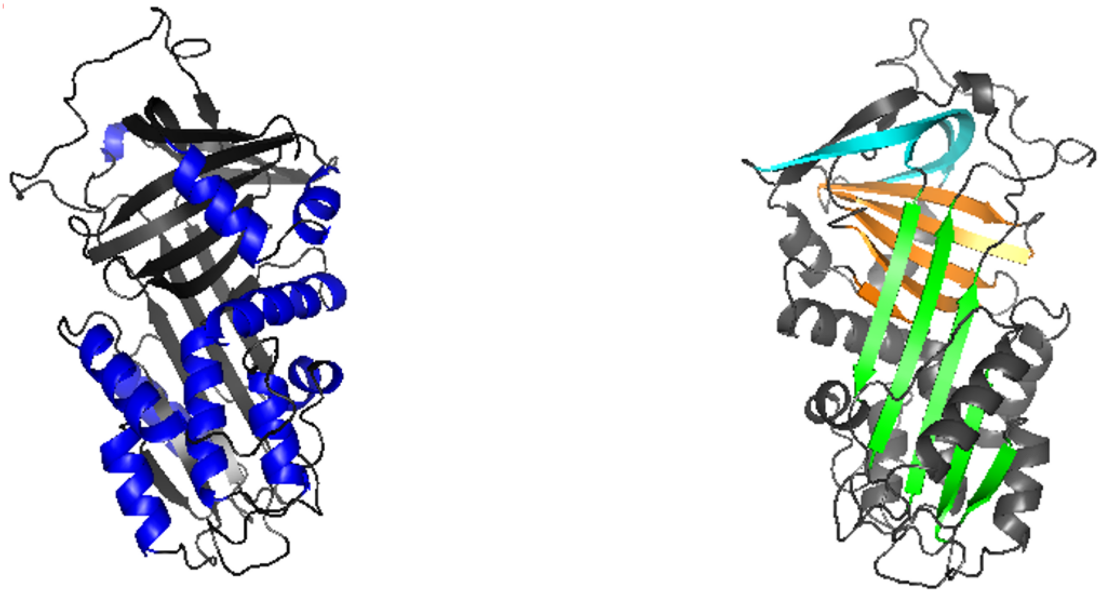

(a) (b)

Figure S2. Cartoon representations of the secondary elements in the active model of Dromaserpin. (a) Overall structure of Dromaserpin model adopting a typical serpin fold composed of 8 α-helices colored blue. (b) Rotation 180 ° left of (**a**) highlighting 3 large β-sheets: β-sheet A is green, β-sheet B is orange, β-sheet C is cyan. Loops are colored grey.

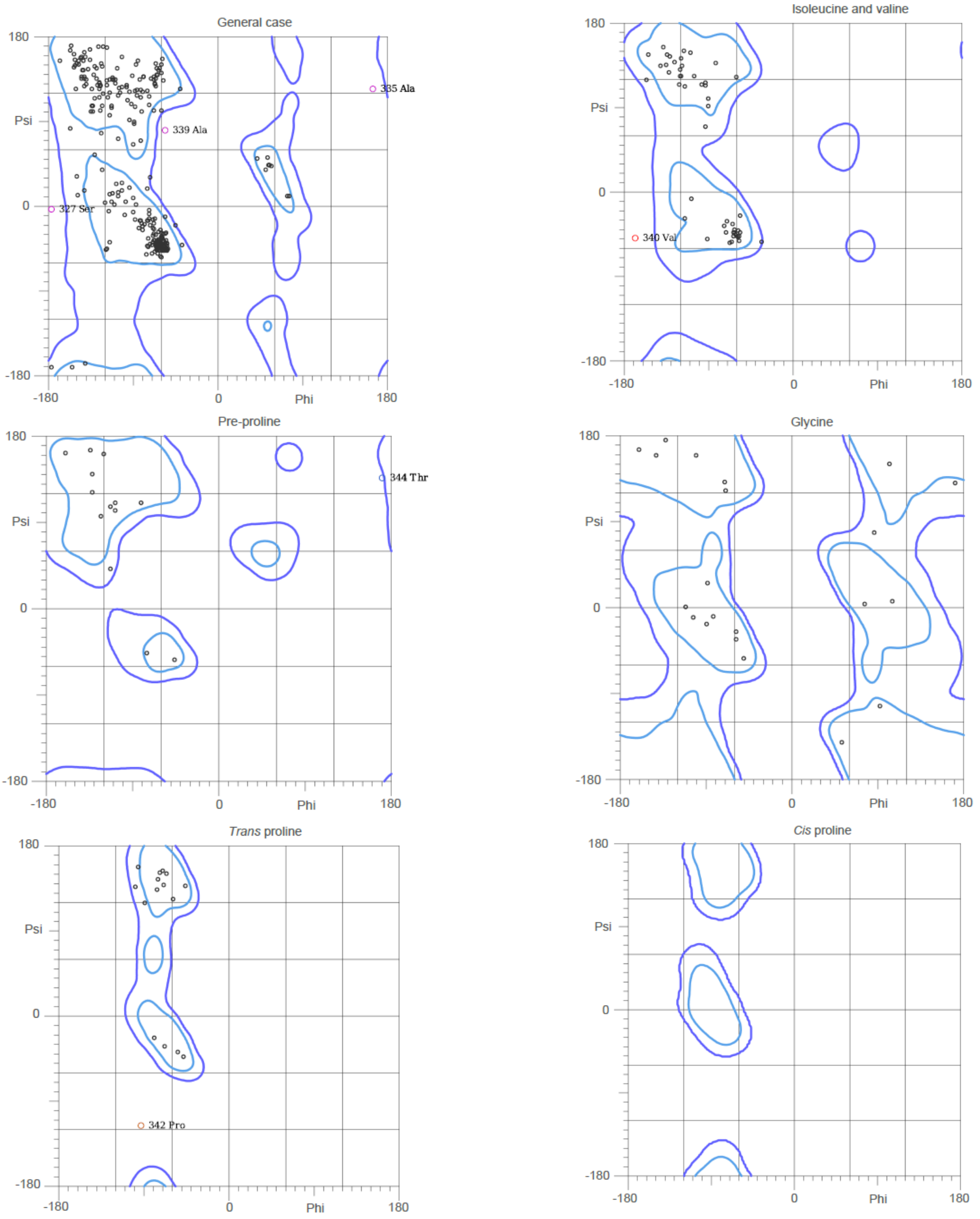

Figure S3. Ramachandran plot of Dromaserpin Model with an exposed reactive center loop (RCL) generated by MolProbity program. 91.9% (340/370) of all residues were in favored (98%) regions. 98.4% (364/370) of all residues were in allowed (>99.8%) regions. There were 6 outliers (phi, psi): 327 Ser (−177.1, −3.9), 335 Ala (165.3, 125.3), 339 Ala (−56.3, 81.9), 340 Val (−169.4, -49.3), 342 Pro (−93.7, −116.9), and 344 Thr (171.9, 137.7).

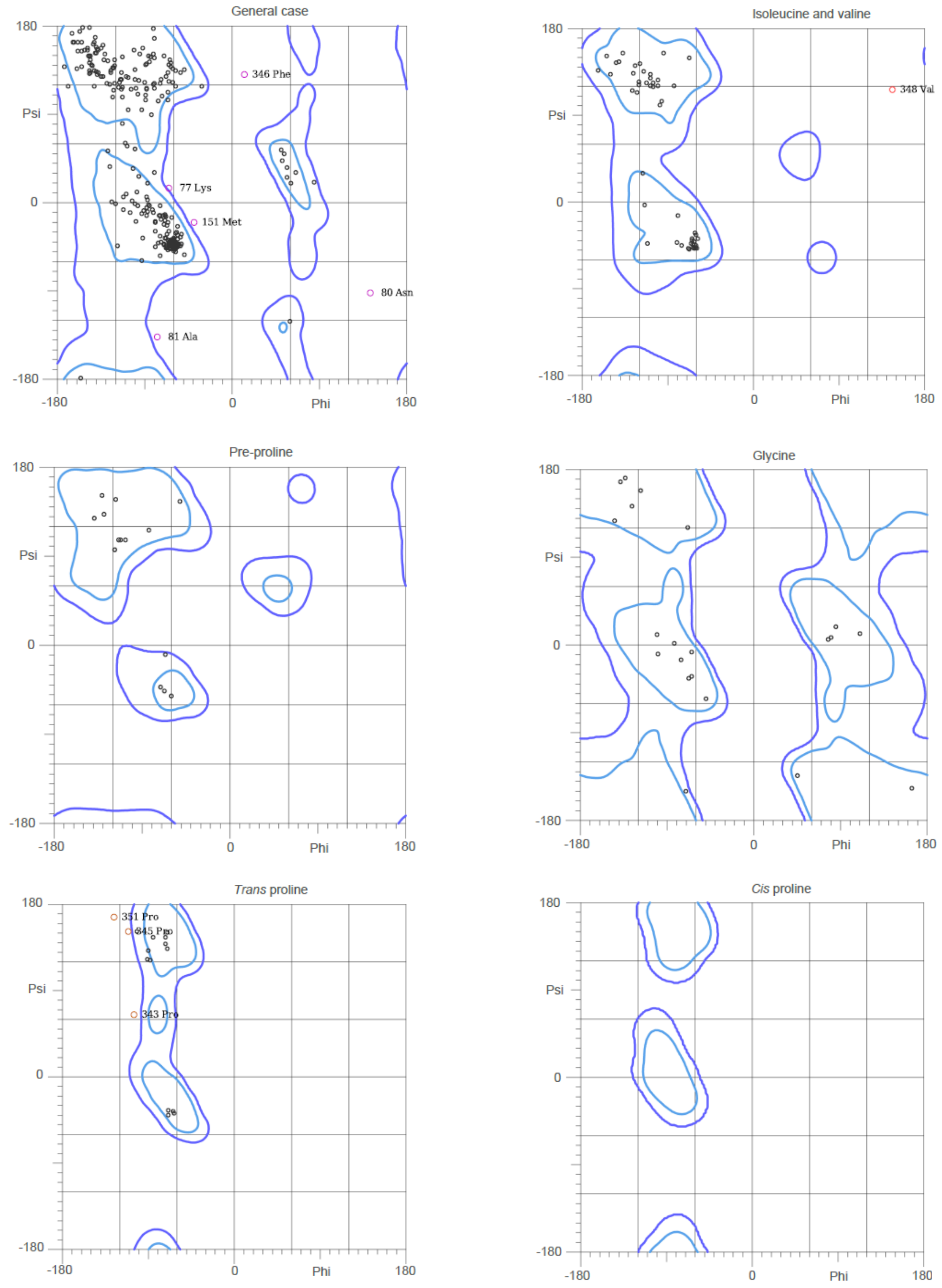

Figure S4. Ramachandran plot of Dromaserpin Model with an inserted RCL generated by MolProbity program. 92.2% (341/370) of all residues were in favored (98%) regions. 97.6% (361/370) of all residues were in allowed (>99.8%) regions. There were 9 outliers (phi, psi): 77 Lys (−65.9, 15.3), 80 Asn (143.2, −92.8), 81 Ala (−77.7, −137.1), 151 Met (−39.8, −20.3), 343 Pro (−105.9, 66.0), 345 Pro (−111.8, 152.5), 346 Phe (13.1, 131.3), 348 Val (146.9, 117.8), and 351 Pro (−126.6, 167.7).

References

32. Yu, Y.; Cao, J.; Zhou, Y.; Zhang, H.; Zhou, J. Isolation and characterization of two novel serpins from the tick *Rhipicephalus haemaphysaloides*. *Ticks Tick Borne Dis.* **2013**, *4*, 297–303. https://doi.org/10.1016/j.ttbdis.2013.02.001.

34. Tirloni, L.; Seixas, A.; Mulenga, A.; Vaz, I. da S.; Termignoni, C. A Family of serine protease inhibitors (serpins) in the cattle tick *Rhipicephalus (boophilus) microplus*. *Exp. Parasitol.* **2014**, *137*, 25–34. https://doi.org/10.1016/j.exppara.2013.12.001.
47. Wang, F.; Song, Z.; Chen, J.; Wu, Q.; Zhou, X.; Ni, X.; Dai, J. The immunosuppressive functions of two novel tick serpins, HlSerpin-a and HlSerpin-b, from *Haemaphysalis longicornis*. *Immunology* **2019**, *159*, 109–120. https://doi.org/10.1111/imm.13130.
50. Chmelar, J.; Oliveira, C.J.; Rezacova, P.; Francischetti, I.M.B.; Kovarova, Z.; Pejler, G.; Kopacek, P.; Ribeiro, J.M.C.; Mares, M.; Kopecky, J.; et al. A Tick salivary protein targets cathepsin G and chymase and inhibits host inflammation and platelet aggregation. *Blood* **2011**, *117*, 736–744. https://doi.org/10.1182/blood-2010-06-293241.
71. Bock, S.C.; Wion, K.L.; Vehar, G.A.; Lawn, R.M. Cloning and Expression of the cDNA for Human Antithrombin III. *Nucleic Acids Res* **1982**, *10*, 8113–8125, doi:10.1093/nar/10.24.8113.
72. Mulenga, A.; Khumthong, R.; Blandon, M.A. Molecular and Expression Analysis of a Family of the *Amblyomma americanum* Tick Lospins. *J Exp Biol* **2007**, *210*, 3188–3198, doi:10.1242/jeb.006494.
73. Porter, L.; Radulović, Ž.; Kim, T.; Braz, G.R.C.; Da Silva Vaz, I.; Mulenga, A. Bioinformatic Analyses of Male and Female *Amblyomma americanum* Tick Expressed Serine Protease Inhibitors (Serpins). *Ticks Tick Borne Dis* **2015**, *6*, 16–30, doi:10.1016/j.ttbdis.2014.08.002.
74. Imamura, S.; da Silva Vaz Junior, I.; Sugino, M.; Ohashi, K.; Onuma, M. A Serine Protease Inhibitor (Serpin) from *Haemaphysalis longicornis* as an Anti-Tick Vaccine. *Vaccine* **2005**, *23*, 1301–1311, doi:10.1016/j.vaccine.2004.08.041.
75. Chlastáková, A.; Kotál, J.; Beránková, Z.; Kaščáková, B.; Martins, L.A.; Langhansová, H.; Prudnikova, T.; Ederová, M.; Kutá Smatanová, I.; Kotsyfakis, M.; et al. Iripin-3, a New Salivary Protein Isolated From *Ixodes ricinus* Ticks, Displays Immunomodulatory and Anti-Hemostatic Properties In Vitro. *Front Immunol* **2021**, *12*, 626200, doi:10.3389/fimmu.2021.626200.
76. Schwarz, A.; von Reumont, B.M.; Erhart, J.; Chagas, A.C.; Ribeiro, J.M.C.; Kotsyfakis, M. De Novo *Ixodes ricinus* Salivary Gland Transcriptome Analysis Using Two Next-Generation Sequencing Methodologies. *FASEB J.* **2013**, *27*, 4745–4756, doi:10.1096/fj.13-232140.
77. Ibelli, A.M.G.; Kim, T.K.; Hill, C.C.; Lewis, L.A.; Bakshi, M.; Miller, S.; Porter, L.; Mulenga, A. A Blood Meal-Induced *Ixodes scapularis* Tick Saliva Serpin Inhibits Trypsin and Thrombin, and Interferes with Platelet Aggregation and Blood Clotting. *Int. J. Parasitol.* **2014**, *44*, 369–379, doi:10.1016/j.ijpara.2014.01.010.
78. Mulenga, A.; Tsuda, A.; Onuma, M.; Sugimoto, C. Four Serine Proteinase Inhibitors (Serpin) from the Brown Ear Tick, *Rhiphicephalus appendiculatus*; cDNA Cloning and Preliminary Characterization. *Insect Biochem Mol Biol* **2003**, *33*, 267–276, doi:10.1016/s0965-1748(02)00240-0.
